# Optimal Timing for Post-Activation Potentiation in Women Collegiate Volleyball Players

**DOI:** 10.3390/sports4020027

**Published:** 2016-05-06

**Authors:** Robert Ah Sue, Kent J. Adams, Mark DeBeliso

**Affiliations:** 1 Department of Physical Education and Human Performance, Southern Utah University, Cedar City, UT 84720, USA; robertahsue@gmail.com; 2 Kinesiology Department California State University Monterey Bay, Seaside, CA 93955, USA; kadams@csumb.edu

**Keywords:** back squat, standing long jump, post-activation potentiation (PAP)

## Abstract

Post-activation potentiation (PAP) has been shown to acutely amplify muscular power output and may be advantageous for athletes looking to improve performance. PAP may have an acute window of effectiveness between 2 to 20 min. With correct timing and implementation it may be possible to induce PAP in competitive situations. The purpose of this study was to examine the time frame of potentiation following a PAP warm-up in collegiate female volleyball players. In this study, nine female collegiate volleyball players completed three laboratory sessions over the course of 10 days. During the first session, the athlete’s 5-RM back squat was determined for subsequent use as the conditioning activity to initiate PAP. A repeated measures experimental design was then employed for Sessions 2 and 3 where half of the participants alternately performed either a dynamic or PAP warm-up prior to performing a standing long jump (SLJ) at 2, 6, 10, 14, and 18 min. A mixed-factor repeated measures ANOVA was used to determine the effects of the two warm-up strategies (PAP *vs.* dynamic) on standing long jump (SLJ) performance across time. There was a significant effect for time (*p* < 0.01) and warm-up strategy (*p* < 0.01). Bonferroni *post hoc* techniques determined that the SLJs that followed the PAP warm-up were significantly greater at 2 (4.8%), 6 (3.6%), and 10 (3.6%) min compared to SLJs post-dynamic warm-up (*p* < 0.05). However, those differences did not persist at 14 or 18 min (*p* > 0.05). Further analysis included non-parametric pairwise comparisons (Wilcoxon signed-rank tests) between the SLJ scores at 2, 6, 10, 14, and 18 min (PAP *vs.* dynamic). The non-parametric results were consistent with the parametric results. Within the parameters of this study, it is concluded that performing a 5-RM back squat induces a measureable PAP effect for up to 10 min.

## 1. Introduction

Evidence suggests that muscular power output can be increased by inducing a post-activation potentiation (PAP) effect with heavy resistance exercises (e.g., 5-RM back squat) [1,2,3,4,5,6]. PAP is defined as an increase in muscle performance after a conditioning contraction [7]. More specifically, PAP is an increase in muscle isometric twitch and low frequency titanic force following a conditioning activity [8]. With the appropriate conditioning activity and correct timing of the subsequent athletic action, it may be possible to induce PAP in competitive situations. However, when attempting to apply PAP to improve performance in a competitive environment, the interplay between stimulus and fatigue must be considered. With that said, a practical challenge in using PAP is to determine the optimal conditions in which stimulus and fatigue can coexist to benefit the athlete’s performance [9]. Specifically, if the PAP conditioning activity is too great (volume and/or intensity), or if there is not sufficient recovery time following the conditioning activity, the associated fatigue of the conditioning activity may not be measurably expressed.

Despite evidence supporting the effectiveness of PAP for increasing power output, finding the precise time window of effectiveness post-PAP remains vague [1,2,3,4,5]. Identifying the window of time that muscular power is increased from PAP is a key next step in making PAP principles more applicable to use in an applied setting. In doing so, it is essential to consider two possible reasons for muscular power decline: decay of the physiological mechanisms responsible for PAP and increasing levels of muscular fatigue. Following a maximal strength exercise the effects of PAP will eventually diminish, which makes the specifics of timing that much more important [8,10]. Studies that show no increase in jumping performance often have multiple measuring points within 4 min, which suggest fatigue as a potential contributor [8,11]. An intense activity that might activate a high degree of PAP result might also produce greater fatigue [8]. Suggested modalities of the PAP as a conditioning activity include the back squat and the bench press at intensities ranging from 60%–90% of 1-RM [12]. The National Strength and Conditioning Association (NSCA) suggests that elite athletes may experience positive muscle enhancement from 2 to 20 min following a PAP conditioning activity and that PAP should be reserved for resistance-trained power athletes with high relative strength [12]. Hence, indicating a large variability in the period of a potentiating effect (coexistence with fatigue), which is dependent on the potentiating modality, intensity employed, the potentiated exercise/movement, training status, relative strength, and rest period following the PAP event.

The purpose of this study was to examine the time frame of potentiation following a PAP warm-up in collegiate female volleyball players. We hypothesized that, when athletes used a warm-up (WU) that included a PAP stimulus, they would perform better in the standing long jump (SLJ) than when the athletes used only a dynamic WU. Additionally, it was of primary importance to determine how long the PAP WU advantage lasted.

## 2. Materials and Methods

### 2.1. Participants

Participants were a convenience sample of female volleyball athletes at Salt Lake Community College, Utah (SLCC). Permission from the head coach and school administration was granted prior to the study. Student athletes were asked to volunteer for the study. Permission from the Institutional Review Board to use human subjects was obtained before conducting any training or assessments of the student athlete participants. Participants were given a written consent to read and sign before any action in the study was taken. All athletes were 18 years or older. Table 1 provides participant descriptive information.

### 2.2. Instruments and Apparatus

**Figure 1:**
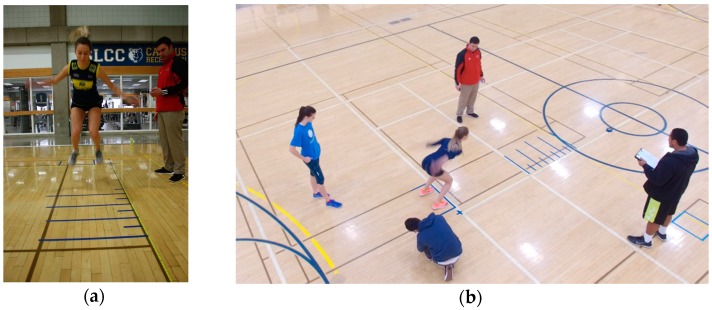
(**a**) Athlete attempting a standing long jump; (**b**) Set up of standing long jump area adjacent to the weight room where 5-RM was performed.

All sessions were held in the Lifetime Activities Center at SLCC. Equipment necessary to conduct this study included a squat rack, a 20.45 kg barbell, weighted plates (ranging from 1.14 to 20.45 kg), measuring tape, blue painter masking tape to set up jump markers (see Figure 1), and a measuring stick.

### 2.3. Procedures

#### 2.3.1. Assessment

**Figure 2:**
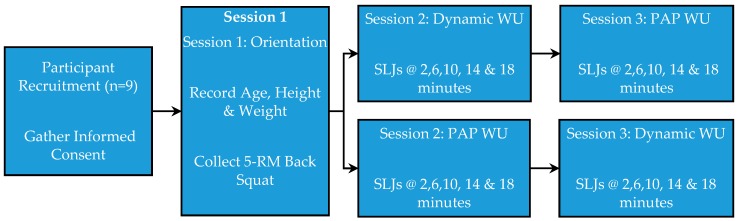
Study time line overview of events. PAP: post-activation potentiation; WU: warm-up; 5-RM back squat used as the PAP WU conditioning activity.

Session 1 consisted of a 45-min study orientation. Participant age, height, and weight were recorded. The investigator then reviewed the proper technique for the execution of the back squat and SLJ with the participants. Following the orientation, the 5-RM back squat was assessed per the recommendations of Baechle and Earle [13] as described below. The 5-RM back squat would later serve as the conditioning activity to induce PAP (modality and intensity) during Sessions 2 and 3 (Figure 2).

Sessions 2 and 3 were held on two consecutive days. During Session 2, all athletes performed the identical dynamic warm-up protocol and then were randomly divided into two groups: PAP and non-PAP. The participants in the non-PAP group rested for 5 min and then proceeded to have SLJs recorded. Those in the PAP group performed back squat sets culminating in a 5-RM set as described below. Two minutes following the 5-RM back squat, the participants started the SLJ trials. During Session 3, the participants crossed-over with regard to the WU procedures, and SLJs were again recorded (Figure 2). The study utilized a randomized repeated measures cross-over design in order to guard against the effects of learning (or testing), which is known to be a threat to internal validity. Further, repeated measures designs are more economical with regard to participant numbers and statistical power.

In order to simulate an “active” athletic setting, instead of a seated rest between jumps, athletes alternated between walking and jogging the length of the basketball court. Thirty seconds prior to their jump, athletes were notified to prepare for their SLJ. SLJs were strictly regulated to the 5 established time markers (2, 6, 10, 14, and 18 min).

#### 2.3.2. Dynamic Warm-Up

The dynamic WU protocol was identical to the WU protocol applied in the athlete’s regular season strength and condition program; this helped maintain consistency/specificity. The standard warm-up consisted of (in specific order): 20 high knees, 20 buttock kickers, 20 lunges, 20 karaoke, 20 A-skips, 20 side shuffle, 30 s of line hops (both feet), 15 s of line hops (one set each leg), 10 broad jumps, 10 squat hops, 10 leg sweeps (hamstring warm up), 20 reverse lunges, 20 walking quad/hamstring stretch, 20 straight leg bounding, 10 leg swings, 10 medium arm circles, and 10 large arm circles.

#### 2.3.3. Standing Long Jump (SLJ)

Athletes started in a standstill position with toes just behind the starting line [13]. The athlete then performed a countermovement and jumped forward as far as possible [13]. The athlete was required to land on their feet for the jump to be scored; otherwise, the trial was repeated [13]. In the event of a fault, the athlete was allowed only one immediate re-jump to avoid fatigue or hindrance of another athlete’s timing. A mark was placed at the back edge of the athlete’s rear most heel, and the tape measure determined the distance between the starting line and the mark [13]. The standing long jump has been shown to be a reliable field test for estimation of muscular power of the lower limbs (ICC = 0.95) [14].

#### 2.3.4. PAP Warm-Up

Athletes performed three warm-up sets of back squats in 8 min (8–10 receptions @ unloaded Olympic bar, 6–8 repetitions @ 30% 1-RM, and 5 repetitions @ 50% 1-RM). Following the WU back squats, athletes were instructed to perform 1 set of 5 repetitions of the back squat using their 5-RM weight (considered the PAP conditioning activity). The reliability of the 1-RM back squat has been reported as ICC = 0.91–0.99 [15].

#### 2.3.5. Dynamic Warm-Up (Non-PAP) Trials

Following the standard warm-up the non-PAP group went directly into SLJ measurements at 2, 6, 10, 14, and 18 min intervals.

#### 2.3.6. Post-Activation Potentiation (PAP) Warm-Up Trials

Following the standard warm-up, the PAP group then performed 3 sets of warm-up back squats followed by 1 set of 5 repetitions of back squats using their current 5-RM. Athletes then performed SLJs on the basketball court (10 feet adjacent from the weight room, Figure 1) at 2, 6, 10, 14, and 18 min intervals. For the purpose of contrasting the results of this study, 1-RM back squat estimates were calculated using the Brzycki equation [16].

#### 2.3.7. Design and Analysis

A mixed-factor repeated measures ANOVA (2X5) was used to determine the effects of the 2 WU strategies (PAP *vs.* dynamic) on SLJ performance at 5 points in time (2, 6, 10, 14, and 18 min). *Post hoc* procedures included single-factor repeated measures ANOVAs and Bonferroni technique paired *t*-tests. ICCs were calculated to determine reliability of the SLJ measures. Effect sizes were also calculated as Cohen’s pair wise effect size (d) = (mean PAP WU SLJ-mean Non-PAP WU SLJ)/Non-PAP WU SLJ SD. Further statistical analysis included non-parametric pairwise Wilcoxon signed-rank tests between the SLJ scores at 2, 6, 10, 14, and 18 min between the two warm-up strategies (PAP *vs.* dynamic). Statistical analysis was conducted using IBM SPSS software (version 24).

## 3. Results

There was a significant effect for time (*p* < 0.01) and warm-up strategy (*p* < 0.01). Repeated ANOVA measures indicated that the SLJs were not significantly different at any point in time following the PAP WU (*p* > 0.05), whereas the SLJs following the Dynamic WU (non-PAP) did vary significantly over time (*p* < 0.01). Bonferroni *post hoc* techniques (paired *t*-tests) determined that the SLJs that followed the PAP WU were significantly greater at 2, 6, and 10 min when compared to the SLJ scores following the dynamic WU (*p* < 0.05). However, those differences did not persist at 14 or 18 min (*p* > 0.05) (see Table 2). It should be noted that the ICC for the SLJ trials following the dynamic WU recorded during the current study was ICC = 0.88 (90% CI: 0.75–0.96).

Due to the small sample size and the potential violations of normality, a nonparametric test was conducted between SLJs at each measurement time interval using the Wilcoxon signed-rank test, as recommended in [17]. The Wilcoxon signed-rank test provides a comparison of the difference in median scores between paired trails, whereas the paired *t*-test compares mean scores between paired trails. The results of the pairwise Wilcoxon signed-rank tests were as follows: 2 min: *p* < 0.05, 6 min: *p* = 0.011, 10 min: *p* = 0.043, 14 min: *p* = 0.26, and 18 min: *p* = 0.12—consistent with the initial statistical analysis.

## 4. Discussion

The objective of this study was to determine if a conditioning activity consisting of a single 5-RM set of the back squat was effective at inducing PAP and increasing SLJ performance and, if so, for what duration. SLJs were performed following two WU scenarios (dynamic and PAP). The SLJs that followed the PAP warm-up were significantly greater at 2, 6, and 10 min when compared to the SLJ scores following the non-PAP warm-up (effect size range 0.70–0.90 SD). However, those differences did not persist at 14 or 18 min. These data suggest that use of a single 5-RM set of the back squat induced PAP and increased SLJ for up to 10 min following the lift (*i.e.*, the conditioning activity). Jumps increased in each of the first three points in time (2, 6, and 10 min) before decreasing, suggesting that the optimal window of time for utilizing the PAP is around 10 min. With that said, the SLJs following the PAP stimuli were on average greater at all time intervals, ranging from 1.5%–4.8%. Hence, the data collected supported the study hypothesis at time intervals 2, 6, and 10 min, but not at 14 or 18 min.

The 5-RM back squats recorded during this study were equated to 1-RM estimates using the Brzycki equation [16]. The 1-RM estimates were 70.6 ± 12.5 kg which is approximately the 60th percentile for NCAA Division I female volleyball players [18]. Multiple studies have used heavy-load back squats as a conditioning activity to increase muscle power through PAP [1,2,3,4,19]. Kilduff *et al.* examined the effects of PAP on a counter jump movement and ballistic bench throws in sample of professional rugby players (*n* = 23) and reported that 8–12 min of recovery time was required between the conditioning activity and the subsequent explosive activity in order to enhance muscle power output [4]. Jenson and Ebben [11] examined the effects of PAP on vertical jump (VJ) performance in a sample of NCAA Division I male and female athletes (*n* = 21) at 10 s and 1, 2, 3, and 4 min following a 5-RM back squat and found decreased performance. In a study comparing VJs at 15 s, 2, 4, 6, 8, 10, 12, 14, and 16 min following a 5 repetition deadlift at 85% of 1-RM, Arias *et al.* demonstrated that PAP was not measurably induced [20]. The data from the Arias study suggest that a high frequency of jumps following a heavy-load conditioning activity may impede PAP [20]. Further, the Arias study used relatively untrained young men (*n* = 15), where training status was suspected to have interfered with the expression of PAP. Ebben suggested improved performance might require 3 to 4 min of rest between the use of a heavyweight training load and plyometrics [21]. In terms of optimal recovery for PAP, this study showed the highest SLJ at 10 min following the back squat. Kilduff *et al.* noted optimal recovery time required for enhanced muscle performance was also in the 8–12 min window [4]. This study, along with previous literature, suggests minimizing fatigue in the first 4 min, and a recovery period of at least 8 min will capture the benefits of an induced PAP more efficiently [1,2,3,4,5,11,21]. It is also important to note that a dynamic warm-up was used prior to the PAP stimulus used in the current study (*i.e.*, a 5-RM back squat). Dynamic warm-ups are shown to enhance performance and should be considered along with PAP [22].

Data suggest that PAP manifests to a greater degree in athletes and highly trained individuals compared to recreationally trained individuals [13,23,24,25]. The NSCA recommends that PAP should be reserved for those with “very high relative strength” [12]. Participants in this study were collegiate female athletes. However, the athlete’s strength to body mass ratios were on average 1.03 kg Est 1-RM back squat/kg body mass, which cannot be considered high relative strength. This is the third such study that has demonstrated that PAP can be effective in individuals with lower relative body strength [26,27]. However, we acknowledge that the effects of the PAP stimulus used in this study may be amplified in participants with higher relative strength. One study comparing PAP in female NCAA athletes called for individual strength considerations along with specific rest times for optimizing power [28]. Xenofondos *et al.* and others suggest that PAP is affected by the training background of individuals and not their gender [7,13]. This suggests the procedures applied in this study may be relevant to male athletes as well. In terms of specific muscle composition, the proportion of fast-twitch motor units might also be related to the effectiveness of neuromuscular activation [7,29]. Again, the participants in this study were collegiate volleyball players, suggesting they have proficient jumping skills, which is typically due to the presence of a higher proportion of fast-twitch motor units. It is vital to contemplate the variety of factors that can affect PAP (training status, potentiating stimulus, fatigue, rest period, window of excitation, muscle fiber composition, *etc.*). As such, we suggest that strength and conditioning coaches follow an individualized approach to implementing PAP training protocols.

In the current study, eight of the nine participants experienced a measureable potentiation as measured by the SLJ as a result of the PAP WU. Following the SLJ assessment collected at the 10 min interval, seven of the nine participants had lower SLJ scores. During the initial 10 min, it appears that fatigue and potentiation coexisted (exhibited by the significantly greater SLJ measures). Fatigue was likely the main contributor to the decrease in SLJs at the 14- and 18-min trials. A recent study examined the effects of PAP (conditioning activity was 3 repetitions of the back squat at 90% 1-RM) on VJ performance in female division II NCAA athletes [28]. VJ performance was assessed prior to, and 5 min after, the PAP back squat set. Collectively, the athletes did not experience a measureable PAP effect. Further, a subset of the participants was volleyball players, and they experienced a reduction in VJ performance. Clearly, fatigue and potentiation did not coexist in this study [28]. We found a comparison of the current study with Sygulla and Fountaine’s study [28] to be revealing. Participants in both studies were female collegiate volleyball players (non-division I), approximately the same age (18–19 years), with similar relative body strength (1.03–1.20 1-RM back squat kg/kg body mass). Conversely, the current study used a 5-RM back squat followed by SLJs initiated 2 min after the PAP conditioning activity, whereas Sygulla and Fountaine’s study [28] used a back squat set of three repetitions at 90% 1-RM followed by VJs initiated 5 min post-PAP conditioning activity. Yet, the results of Sygulla and Fountaine’s study [28] are in direct contrast to the results of our study in regard to jumping performance. It is possible that their study design [28], which measured pre- and post-PAP VJs on the same day in a non-cross over design, is responsible for the discrepant results. Specifically, based on the results and their study design, there is no way to tell if the pre-PAP VJs confounded the effects of the PAP stimulus (conditioning activity). If Sygulla and Fountaine’s study had used a same-day cross-over study design or assessed PAP and non-PAP WUs on separate days, their results may have been more consistent with our study. While the current study did employ a cross-over design, an obvious limitation was the small sample size of *n* = 9. The current study used an intact team of nine female collegiate volleyball players, and the team only had nine players at the time of the study. A future study should attempt to replicate the findings of the current study; otherwise, the results of the current study should be included in a subsequent meta-analysis.

The improvement in the SLJ noted in the current study as a result of the PAP WU is consistent with previous studies that have demonstrated that a PAP WU can significantly increase both upper and lower body power output [26,27,30,31,32,33,34,35]. The SLJ is a full-body coordinated movement driven by lower-body muscle power. It is not certain that the improvements in SLJ ability noted in this study as a result of a PAP WU would be specific and transferable to the unpredictable factors in volleyball competition. However, it is reasonable to think that an elevated physical state induced by a PAP WU (*i.e.*, conditioning activity) may well lead to a slight edge over the competition for the early minutes of a volleyball contest. With that said, a PAP WU strategy might be better utilized when applied as a conditioning activity for predictable performance tests. For example, every year NCAA eligible athletes compete in the NFL combine for an assessment of their athletic ability [36]. The SLJ and VJ are used as an overall indicator of power in these athletes [13,36]. Athletes competing in such assessments could potentially use heavy back squats to induce PAP and improve performance. Long jumpers and triple jumpers are also examples of athletes looking for acute enhancement of muscular power output in predictable competition scenarios. Future research should focus on the effects of a PAP WU on specific sport competitions such as the long jump, the triple jump, and various throwing events.

## 5. Conclusions

Within the parameters of this study: (1) There was an improvement in SLJ following a conditioning activity that combined a dynamic warm-up with a 5-RM back squat; (2) the effects of PAP resulted in significant improvement in SLJ for up to 10 min; (3) therefore, the results of this study suggest that, if the conditioning activity is performed correctly per the specific applied context, PAP may be used to improve power in predictable situations. We suggest further research should be geared towards implementation of PAP in competitive situations.

## Figures and Tables

**Table 1:** Participant descriptive information *.

*N*	Age (years)	Height (cm)	Mass (kg)	5-RM BSQ (kg)	Est 1-RM BSQ (kg)
9	18.3 ± 0.5	179.2 ± 5.3	68.8 ± 7.9	62.9 ± 11.1	70.6 ± 12.5

* Participant means and standard deviations for descriptive information; BSQ-back squat; 5-RM-five repetition maximum; Est 1-RM BSQ-estimated one repetition maximum (Brzycki equation).

**Table 2:** Standing long jump (SLJ) performance at 5 points in time following PAP and Non-PAP warm-ups.

PAP *vs*. Non-PAP SLJ					
	2 min	6 min	10 min	14 min	18 min
Non-PAP WU (cm)	191.4 ± 10.1	196.1 ± 10.2	199.9 ± 10.3	199.6 ± 11.8	200.7 ± 9.6
PAP WU (cm)	200.5 ± 13.9 *	203.3 ± 14.1 *	207.1 ± 13.3 *	202.6 ± 13.5	205.2 ± 13.4
%∆	4.8%	3.6%	3.6%	1.5%	2.2%
*p*-value	0.02	0.03	0.04	0.40	0.09
Effect Size (ES)	0.90	0.71	0.70	0.25	0.47

Participant means and standard deviations (SD) for descriptive information; * PAP WU significantly greater than Non-PAP WU (*p* < 0.05). Paired *t*-test *p*-values; %∆ = (PAP WU SLJ-Non-PAP WU SLJ)/Non-PAP WU SLJ.
